# Development of an augmented reality remote maintenance adoption model through qualitative analysis of success factors

**DOI:** 10.1007/s12063-023-00356-1

**Published:** 2023-04-20

**Authors:** Maike Müller, Dirk Stegelmeyer, Rakesh Mishra

**Affiliations:** 1 School of Computing and Engineering, University of Huddersfield, Huddersfield, UK; 2 Institut Für Interdisziplinäre Technik, Frankfurt University of Applied Sciences, Frankfurt am Main, Germany

**Keywords:** Augmented reality, Mobile collaboration, Technology adoption, Remote service, Product–service system

## Abstract

In recent decades, engineering manufacturers have increasingly adopted servitization strategies, making efficient maintenance service delivery a key corporate activity for revenue growth and profitability. Recently, augmented reality remote maintenance (ARRM) has become available and promises service delivery efficiency gains, which is why engineering manufacturers are starting to adopt it. Therefore, the purpose of this paper is to analyze the promoting and inhibiting factors of ARRM adoption in support of industrial service delivery. To this end, a qualitative explorative approach based on the technology–organization–environment (TOE) framework has been deployed. Data were collected from a systematic literature review and an empirical interview study with 16 companies. In total, 38 interviewees from different hierarchical levels participated in the interview study. Based on a thematic analysis, this paper provides a novel ARRM adoption model and contributes six technological, seven organizational, and four environmental adoption success factors. The promoting factors are ARRM key features, qualitative operational advantages, quantitative operational advantages, value proposition, value creation network, finance, image, adoption management, resource allocation, strategic realignment, skills gap, and governmental regulation; further, augmented reality user experience, data connection, information provision, intellectual property protection, and remote service acceptance were identified as inhibiting factors. This paper qualifies as the first attempt to consolidate engineering-focused ARRM literature with respect to remote service delivery, adding the industrial adoption perspective and elaborating on the impact of the inter-organizational nature of ARRM technology on industrial adoption. Thus, this research contributes to the transition from pure engineering research to industrial ARRM adoption research.

**Supplementary Information:**

The online version contains supplementary material available at 10.1007/s12063-023-00356-1.

## Introduction

In response to increasingly competitive capital equipment markets, many equipment manufacturers have servitized their businesses (Dachs et al. 2014; Mastrogiacomo et al. 2020; Ulaga and Reinartz 2011). To this end, equipment manufacturers bundle their capital equipment products (e.g., machine tools, material handling systems, and valve technology) with maintenance services directed to their installed base (e.g., equipment installation, fault diagnosis, repair, overhaul, and application engineering optimization) to provide value to their customers, build revenue, and achieve profitability growth (Oliva and Kallenberg 2003; Wise and Baumgartner 1999). The key quality requirements for such product–service systems are accessibility, locality, and responsiveness of services—i.e., customers expect very short response times to equipment failures (Biehl et al. 2004; Marcon et al. 2022; Toossi et al. 2013). This especially holds true for failures causing downtime in equipment as their unavailability creates huge opportunity costs for customers. Thus, equipment manufacturers are expected to provide reliable and effective services (Allmendinger and Lombreglia 2005; de Jong and Smit 2019). However, equipment manufacturers are confronted with a worldwide distributed installed base of products, which often causes long travel times to troubleshoot at customers’ sites as skilled service experts are a scarce resource and are usually not spread evenly across regions. Therefore, efficient and effective remote service delivery is required—e.g., by cutting out field service technicians’ travels whenever possible.

On these grounds, augmented reality (AR) remote maintenance (ARRM) is one of the most relevant technological trends (Egger and Masood 2020; Palmarini et al. 2018; Silvestri et al. 2020). ARRM is described as a technology that enables multiple users (i.e., remote experts and on-site technicians) who are not in the same physical space to share the same augmented environment to facilitate knowledge transfer and accomplish physical maintenance tasks (Breitkreuz et al. 2022; Fang et al. 2020; Marques et al. 2022). In contrast to other industrial AR applications (e.g., automatic single-user step-by-step assembly guidance), ARRM is not only limited to standardized procedures, but is also suitable for non-standard problems, for example to diagnose failure sources in interdisciplinary teams (Kleiber and Alexander 2011). Moreover, ARRM is suitable for situations in which knowledge is unavailable during on-site service interventions (Marques et al. 2022). Therefore, ARRM is an attractive technology to improve the efficiency and effectiveness of industrial product–service systems.

Because many of the most obstructive technical limitations of ARRM are overcome, the technology seems to be on the verge of becoming a remote service delivery tool in the industry (Masoni et al. 2017; Si2 Partners 2018). Nevertheless, it is still far from being diffused across industry (Jalo et al. 2022; Marques et al. 2022; Runji et al. 2022; Si2 Partners 2018). The reason might be that industry is struggling with organizational rather than technical adoption aspects (Masood and Egger 2019; Si2 Partners 2018). Prior ARRM research has focused on technical innovation and prototype development (Breitkreuz et al. 2022; Marques et al. 2022). Hence, research on organizational and environmental adoption aspects (e.g., alignment of business processes, culture, and business partner readiness) is lacking in the literature (Egger and Masood 2020; Herterich et al. 2015; Masood and Egger 2019). Moreover, in contrast to other industrial AR applications, ARRM is an inter-organizational technology (Mourtzis et al. 2017a; Ohlig et al. 2020; Porter and Heppelmann 2017). That is, it is a shared information system among a group of companies (de Pablos Heredero and de Pablos Heredero 2010). This distinguishing characteristic makes the adoption process more complex because companies external to the adopting equipment manufacturer are to be involved in the adoption process (Kuan and Chau 2001), at least in reference to customers whose equipment is to be serviced. To fully understand ARRM adoption and leverage its potential benefits and opportunities, equipment manufacturers must develop adoption strategies that consider technical and organizational aspects (Porter and Heppelmann 2017). Therefore, a holistic analysis that goes beyond the technical aspects is required and is, hence, presented in this paper.

To initiate research on ARRM adoption and industrial adoption strategies, the aim of this paper is to establish the success factors for industrial ARRM adoption. For this purpose, this paper develops an ARRM adoption model that considers the technological, organizational, and environmental dimensions of the adoption process. This contributes to the literature by providing an ARRM-specific adoption model, and it contributes to practice by providing comprehensive items to be considered during implementation. To develop the model, the following research question was used to guide this study: “What are promoting and inhibiting ARRM adoption factors when considering the specific challenges of industrial product–service systems provision?”

To answer this question, an explorative qualitative methodology utilizing data from a systematic literature review and a qualitative interview study was applied. Our paper offers three major contributions:

- Providing a holistic ARRM-specific adoption model that considers the technological, organizational, and environmental dimensions
- Identifying 17 adoption success factors relevant to the inter-organizational context of product–service systems
- Operationalizing success factors by defining 53 specific items making ARRM adoption success measurable

The remainder of the paper is organized as follows. Section 2 provides a theoretical background regarding the challenges of industrial product–service systems provision, related works, and technology adoption frameworks. In Section 3, the research framework of this study is developed, followed by the methodology in Section 4. The results are then presented in Section 5 and discussed in Section 6. Finally, the conclusion, theoretical and practical contributions, limitations of the research, and further research suggestions are addressed in Section 7.

## Theoretical background

### Challenges of product–service systems provision

Product–service system provisions are often characterized by value creation networks (Gebauer et al. 2010; Marcon et al. 2022; Oliva and Kallenberg 2003), meaning that apart from the equipment manufacturer, the customers whose products are to be serviced must be integrated into the service delivery process. Often, component manufacturers, system integrators, and third-party service providers are also involved (Marcon et al. 2022). Therefore, efficient service delivery depends on the network participants’ ability to diffuse information and knowledge to achieve collaborative troubleshooting (Oliva and Kallenberg 2003).

However, collaborative troubleshooting with customers is challenging because the prevailing practice is still a combination of telephone, email, and paper-based technical support (del Amo et al. 2020; Vorraber et al. 2020), which leads to excessive repair times (Jonsson et al. 2008). This is because communication challenges (e.g., language barriers and different technical terminology) often result in misunderstandings and inaccurate failure identification (Jalo et al. 2018; Jonsson et al. 2008). Moreover, the ever-increasing complexity of products not only implies more potential failure sources but also requires increased specialized information and knowledge (Aschenbrenner et al. 2019; Herterich et al. 2015). However, specialized knowledge is a scarce commodity in equipment manufacturing fields (Marques et al. 2022). Thus, tasking highly skilled engineers with unproductive service calls restricts the capacity urgently required elsewhere.

In cases where remote troubleshooting fails, field service technicians are usually deployed to customer sites to troubleshoot (Brax and Jonsson 2009). However, field technicians require accurate failure identification data in advance so that they can bring the correct tools and parts (Jonsson et al. 2008; Küssel et al. 2000). It is also commonplace for technicians to be deployed based on their immediate availability instead of prioritizing their subject-matter expertise (Küssel et al. 2000; Marques et al. 2022). Moreover, the inevitable growth of pools of less qualified field service technicians increases over time (Herterich et al. 2015). These situations often require secondary and often unpaid deployments, which are costly and tie-up capacity (Küssel et al. 2000; Marques et al. 2022). To address these challenges, equipment manufacturers have adopted various kinds of remote service technologies (e.g., remote access to machine controls and condition monitoring). As noted, ARRM has recently extended the toolbox of available remote service technologies in support of product–service systems.

### Related works and literature gaps

Thus far, ARRM research has focused on developing ever more sophisticated prototypes in laboratory-controlled settings (Breitkreuz et al. 2022; Marques et al. 2022). Thus, industrial ARRM applications are lacking. Presently, only a few industrial ARRM adoption studies are available. Early work was presented by Rapaccini et al. (2014), who investigated organizational adoption challenges and user acceptance issues of an Italian printing equipment manufacturer’s field service unit. Jalo et al. (2022) identified application opportunities and enablers of ARRM adoption based on interviews and focus groups with five Finnish companies. However, they investigated ARRM in terms of supporting facility management, which is a completely different business environment compared to industrial product–service systems.

Other researchers have provided adoption studies on a wide range of industrial AR applications and use cases (Egger and Masood 2020; Jalo et al. 2022; Masood and Egger 2019; Porter and Heppelmann 2017; Si2 Partners 2018), whereas we focus solely on AR in a remote collaborative and inter-organizational troubleshooting setting. Porter and Heppelmann (2017) presented a narrative work discussing the potential of industrial AR using illustrative adoption examples of US firms. Si2 Partners (2018) presented a non-scientific and descriptive consultancy survey on AR adoption in support of industrial service delivery. The insights of these works are very valuable as they addressed inter-organizational industrial service settings and focused strongly on ARRM. However, these studies are not meeting scientific transparency standards with respect to research designs.

Masood and Egger (2019) surveyed implementation challenges and industrial AR adoption factors in a correlation study that utilized qualitative and quantitative methods. However, their study focused on the automotive and transportation industries and included use cases such as picking, navigation, and design. Moreover, they based their adoption model purely on the literature. Hence, their model strongly focused on technical challenges while underrepresenting the effects of organizational and environmental issues. Nevertheless, the qualitative part of their study demonstrated that the industry is indeed struggling with organizational adoption aspects. These researchers further identified the understanding of organizational and environmental factors as a gap in the literature (Egger and Masood 2020; Masood and Egger 2019). These issues are highly relevant to implementation practices as companies seem to routinely underestimate the organizational challenges of ARRM adoption, such as a lack of acceptance, failure to implement the right processes, and mismanagement of change (Si2 Partners 2018).

Jalo et al. (2022) identified 13 enabling factors for extended (augmented and virtual) reality, proposing an adoption model based on an interview study with 45 European manufacturing companies. Their methodology is comparable to ours and contributes to the nascent research on organizational adoption factors. However, their study focused on a wider range of technologies, use cases, and industries compared to ours. Moreover, they investigated manufacturing industries (e.g., textiles, furniture, and food) that do not provide product–service systems, and their scope of work included use cases related to event management and design visualization.

Our review of related studies shows that previous research has focused on a wide range of industrial AR technologies, applications, and use cases. Moreover, the inter-organizational nature of ARRM in support of product–service systems is not reflected in other studies, and organizational and environmental aspects are underrepresented in the literature thus far. Although related adoption studies provide great value, it is yet to be shown if and how these insights are applicable to ARRM. Thus, our study aims to holistically investigate ARRM adoption from combined technological, organizational, and environmental perspectives. Furthermore, we intend to operationalize the adoption success factors specific to inter-organizational product–service system provision.

### Technology adoption models

Generally, technology adoption is the process of implementing technology developed elsewhere (van de Ven et al. 2000). ARRM software is currently provided by a vast number of startup companies (e.g., Oculavis, Fieldbit, and XMReality) as well as established vendors with a background in teleconferencing (e.g., Librestream and TeamViewer) and other fields (e.g., Microsoft and PTC). With respect to hardware, various types of head-mounted displays (HMDs) are commercially available (e.g., RealWear Navigator 500, Vuzix Blade, Epson Moverio BT-35ES, and Microsoft HoloLens 2). Thus, ARRM adoption can be summarized as the process of configuring and implementing these vendor technologies into the equipment manufacturers’ existing information technology (IT) infrastructure, service processes, and offerings.

Over the past seven decades, researchers have provided a multitude of theories and models to explain and predict the technology adoption behavior of individuals, groups, and organizations (Liu et al. 2008). Some of the most prominent include innovation diffusion theory (IDT) (Rogers 2003), the theory of reasoned action (TRA) (Fishbein and Ajzen 1975), the theory of planned behavior (TPB) (Ajzen 1985, 1991), the technology acceptance model (TAM) (Davis 1986), the technology–organization–environment (TOE) framework (DePietro et al. 1990), and the unified theory of acceptance and use of technology (UTAUT) (Venkatesh et al. 2003). However, the TRA, TAM, TPB, and UTAUT focus on the behavior of individuals; thus, these theories are more suited for consumer applications and too limited for organizational AR adoption in industry (Masood and Egger 2019).

While IDT and the TOE framework are consistent with respect to the technology and organizational dimensions, only TOE considers the external environment (Masood and Egger 2019). For inter-organizational technology adoption, the external environment is inevitably a dimension to be considered (Kuan and Chau 2001) as the customers in that domain are to be directly involved in ARRM adoption and operational service provision. Therefore, the TOE framework is preferred over the IDT as a general research framework to guide this study. Additionally, the TOE framework provides a strong empirical basis across related technologies, including industrial AR (Masood and Egger 2019), industrial extended reality (XR) (Jalo et al. 2022), eMaintenance (Aboelmaged 2014), and earlier inter-organizational innovations, such as electronic data interchange (Hsu et al. 2006; Zhu et al. 2003, 2006). However, TOE is rather generic and does not specify the concrete constructs and factors that determine technology adoption success. Therefore, when using the TOE framework, constructs and factors influencing technology adoption need to be adapted to the specific technology and context of the study (Baker 2012). This conceptual adaptation of the TOE framework to the specific context of ARRM adoption is provided in the following section by developing the research framework for this study.

## Research framework

Generally, the TOE framework assumes that organizational technology adoption is affected by three dimensions: technology, organization, and environment. ARRM adoption success is considered achieved when the adopting equipment manufacturer, their customers, and/or their value creation partners use the technology on a regular basis (Damanpour 2016). Thus, success factors are understood as those promoting and inhibiting factors (i.e., benefits and opportunities arising from ARRM, challenges and key activities of adoption projects) that need to be recognized, overcome, and pursued in order to achieve ARRM adoption success. Identifying success factors will help us to better understand why ARRM is not yet widely diffused in industry. Our research framework was developed based on insights from previous TOE studies (e.g. Arnold and Voigt 2019; Kuan and Chau 2001; Masood and Egger 2019; Zhu et al. 2003), and studies of related remote service technology in support of product-service provision; i.e., remote condition monitoring and smart services (e.g. Biehl et al. 2004; Herterich et al. 2015; Klein et al. 2018; Paluch and Wunderlich 2016). We paid particular attention to the specific characteristics of ARRM-based product-service systems, e.g., the inter-organizational nature.

**Fig. 1:**
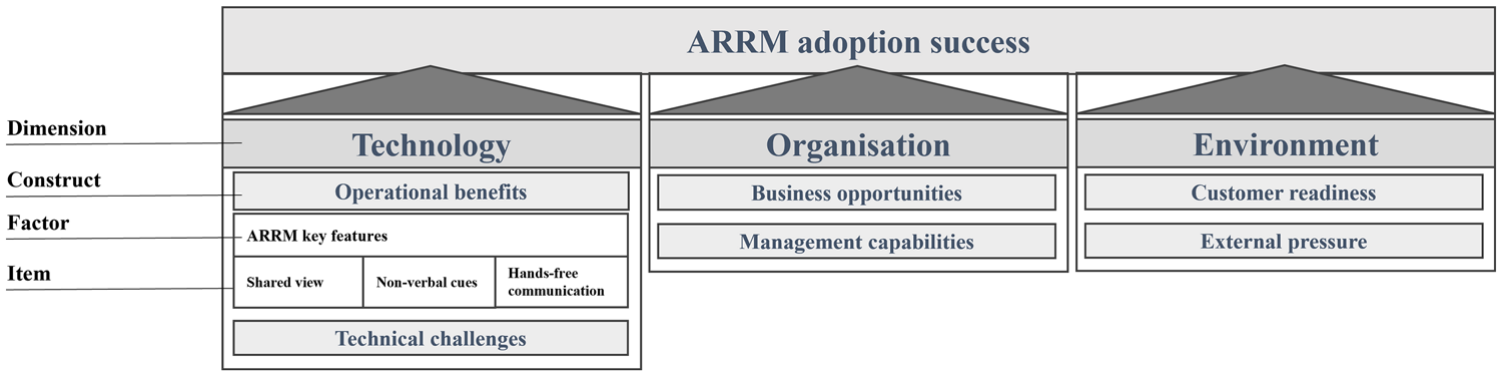
Technology–Organization–Environment-based augmented reality remote maintenance (ARRM) research framework

Each TOE dimension consists of different constructs capturing the promoting or inhibiting factors of technology adoption. The constructs represent the categories for ARRM adoption success factors, while adoption success factors, on the other hand, are conceptualized categories for items—i.e., measurable units. Within this section, the constructs as a general perspective on ARRM adoption are derived, while the factors and items are the results of this study presented in section 5. The research framework of this study is presented in Fig. 1, including an example of factors and items that clarify the terminology used in this paper.

### Technological dimension

The technological dimension represents current practices and equipment already used in the adopting company as well as attributes of the technology under investigation (DePietro et al. 1990). Within the technological dimension, researchers often used technological characteristics—i.e., factors that describe the innovation itself, such as relative advantage, technical challenges, trialability, complexity, and ease of use (Jeyaraj et al. 2006).

#### Operational benefits

Relative advantage is a technological construct that is extensively used in TOE adoption studies and consistently shows a significant effect on technology adoption (Jeyaraj et al. 2006; Mangula et al. 2015). According to Rogers (2003), “relative advantage is the degree to which an innovation is perceived as being better than the idea it supersedes.” Relative advantage was found to be the most influential predictor of industrial internet-of-things adoption (Arnold and Voigt 2019), and it has a significant effect on AR use in e-commerce (Chandra and Kumar 2018) and other inter-organizational technologies (Hsu et al. 2006; Kuan and Chau 2001). However, relative advantage can appear in very different forms (Tornatzky and Klein 1982). Therefore, especially in the context of inter-organizational technology studies, researchers have distinguished operational benefits from business opportunities (Iacovou et al. 1995; Kuan and Chau 2001). For example, Kuan and Chau (2001) referred to operational benefits as the “improvements made to the internal functioning of the organization apparent in everyday activities.” Operational benefits are often viewed as the technology’s key features—i.e., shared views, awareness cues, and hands-free usage for on-site technicians (cf. Figure 1). Other operational benefits associated with remote service technologies include enhanced responsiveness, higher accuracy, fewer errors (Herterich et al. 2015), reduced expert travel, faster fault and spare part identification, and improved preparation of on-site interventions in cases where remote troubleshooting is not feasible (Küssel et al. 2000).

#### Technical challenges

Novel remote service technologies, such as ARRM, often suffer from immaturity and needs to be integrated into the existing IT infrastructure of the adopting organization; in other words, adoption is also associated with technical challenges. Technical challenges, such as the immaturity of HMDs, were found to have a significant negative effect on industrial AR adoption (Masood and Egger 2019). Notably, HMD immaturity is a major adoption barrier as it prevents user acceptance (Jalo et al. 2022; Masood and Egger 2019). Additionally, the integration of other technologies already in use in the adopting company can be rife with common technical challenges, since tracking the service history and providing relevant data is only possible if integration occurs in the existing technological infrastructure (Herterich et al. 2015; Jalo et al. 2018, 2022). Moreover, equipment manufacturers tend to underestimate the efforts needed to overcome technical challenges—e.g., the lack of connectivity at the installed base (Klein et al. 2018; Si2 Partners 2018).

### Organizational dimension

The organizational dimension captures the characteristics of the adopting organization’s ability to promote or prevent technology implementation (DePietro et al. 1990). Within this dimension, researchers often use constructs related to managerial aspects, firm size, organizational challenges, and expected benefits.

#### Business opportunities

While operational benefits (cf. the technological dimension) are directly linked to ARRM, business opportunities are rather indirectly beneficial to the organization’s strategic positioning (Iacovou et al. 1995). Examples of business opportunities include improvements with regard to competitive advantages, image, or relationships with customers and other business partners (Kuan and Chau 2001). Business opportunities commonly associated with remote service technologies include cost reduction, increased service quality, minimized installed base downtime, expanded service offerings, and additional revenue (Grubic 2014; Herterich et al. 2015; Küssel et al. 2000). Moreover, a global service network performing high service levels is becoming archivable for smaller manufacturers lacking necessary resources too (Biehl et al. 2004; Küssel et al. 2000).

#### Management capabilities

Top-management support refers to senior executive commitment to technology adoption, which is particularly expressed by appropriate resource allocation (Mangula et al. 2015) and is an extensively used construct in previous TOE studies (e.g., Sun et al. 2018; Chandra and Kumar 2018; Oliveira et al. 2014). The construct is considered particularly important in inter-organizational technology adoption (Grover 1993), is generally among the best predictors of IT adoption (Jeyaraj et al. 2006; Mangula et al. 2015), and was recently identified as an factor enabling industrial AR adoption (Jalo et al. 2022).

Apart from top management support, managerial obstacles (Zhu et al. 2006) and organizational fit (Masood and Egger 2019) have shown significant effects in related studies. Managerial obstacles and organizational fit refer to an organization’s ability to reengineer business processes, align technology with strategic goals, acquire the expertise necessary for implementation and operations, and involve users early in the implementation journey. The latter item (i.e., involving field service technicians and remote experts) is particularly important because technological immaturity and mistrust in ARRM can negatively affect user acceptance (Masood and Egger 2019). Additionally, the high fragmentation of hardware and software solutions available on the market complicates ARRM technology configuration (Jalo et al. 2022; Palmarini et al. 2018). Moreover, equipment manufacturers tend to underestimate the managerial capabilities required for ARRM adoption—e.g., addressing the lack of user acceptance, implementing the right processes, managing change, or monitoring adoption progress (Si2 Partners 2018). Therefore, in order to recognize top-management support and other middle management duties within our research framework, we use a combined management capabilities construct that captures top-management support as well as managerial obstacles and organizational fit.

### Environmental dimension

The environmental dimension extends the technology adoption framework beyond the organization’s boundaries, fully considering the ecosystem in which the business is conducted (DePietro et al. 1990). Within the environmental dimension, researchers often assess customer or trading partner readiness, competitive pressure and intensity, governmental regulations, industry standards, and external support from specialized technology vendors and consultancies.

#### Customer readiness

Customers’ willingness to use ARRM capabilities is a decisive factor in adoption success. Because customer readiness seems to be a common barrier to industrial XR adoption, it is likely that ARRM will also be affected (Jalo et al. 2022). In addition, in previous adoption studies of other inter-organizational technologies, customer readiness showed a significant effect on adoption (Oliveira and Martins 2010; Zhu et al. 2003).

Although there is reason to believe that customers will demonstrate high acceptance rates for ARRM (Si2 Partners 2018), customer readiness could still be particularly negatively affected by concerns of losing control over intellectual property (Toossi et al. 2013). As a matter of fact, intellectual property and privacy protection concerns are well-documented barriers to remote service technology adoption (Klein et al. 2018; Paluch and Wunderlich 2016). To counteract these concerns, customers from sensitive industry sectors in some instances even have imposed IT regulations that completely prohibit outgoing data connections, which is a knockout blow to ARRM adoption. Moreover, customers’ individual user acceptance (e.g., maintenance personnel or machine operators) is required for ARRM, since, for some use cases, the customers’ employees are the counterpart of equipment manufacturers’ remote experts (Mourtzis et al. 2017a; Ohlig et al. 2020). Yet, customers’ users might have personal fears such as those related to a decrease in personal contact or self-efficacy doubts and, thus, are averse to using ARRM, just like they are averse to using other remote service technologies (Paluch and Wunderlich 2016). Notably, the appropriate billing of remote services is a common challenge (Klein et al. 2018).

#### External pressure

External pressure to adopt novel technologies refers to influences from market players with whom the adopting organization performs its business—e.g., customers, competitors, suppliers, or governments (Iacovou et al. 1995; Mangula et al. 2015). These pressures are among the best predictors of IT adoption (Jeyaraj et al. 2006; Mangula et al. 2015) and have recently been identified as enabling factors of industrial XR adoption (Jalo et al. 2022). A recent example of external ARRM adoption pressure is the government-mandated travel restrictions imposed during the COVID-19 pandemic, which tremendously hindered international service delivery (Cavaleri et al. 2021; Li et al. 2022; Wuest et al. 2020).

## Research methodology

This study aims to propose a ARRM adoption model establishing the success factors of industrial ARRM adoption; thus, an exploratory qualitative approach has been adopted. For explorative studies, qualitative research is most suitable because it emphasizes hypothesis generation (i.e., research propositions) over hypothesis testing (de Ruyter and Scholl 1998). To develop our model, we asked the following research question, “What are promoting and inhibiting ARRM adoption factors when considering the specific challenges of industrial product–service systems provision?”

To answer this question, a systematic literature review of ARRM research and a qualitative interview study were conducted. Given that ARRM research is still at an early stage, we acknowledge that it retains a strong engineering focus (Breitkreuz et al. 2022). Hence, the results of a literature review alone would lack an industrial organizational basis. Thus, we compensated for the literature review with an interview study with 38 participants from 16 companies. The goal of the interviews was to uncover nontechnical factors that have not yet been addressed in the literature. This ensures the validity of the results for real-world industrial service contexts.

### Data collection

#### Literature review

In prior literature reviews, ARRM and other single-user AR maintenance applications were undifferentiated (Egger and Masood 2020; Masood and Egger 2019; Palmarini et al. 2018; Runji et al. 2022). Because our scope is focused on ARRM in support of product–service systems, we only sought literature on collaborative applications spanning the past two decades, utilizing Scopus and Web of Science search engines (cf. Table 1). Our search string comprised three aspects. First, “augmented reality” describes the core technology used for ARRM; second, “remote collaboration” characterizes the interactions between at least two physically separated users during ARRM sessions; and third, “maintenance context” outlines the industrial application of the relevant technologies. Our search was performed in December 2020, covering articles published in English and German.

**Table 1:** Search terms and filters applied to the database search

Database	Search string	Filter	Articles
Scopus	TITLE-ABS-KEY (“augmented reality” AND (tele* OR remote OR collaborat*) AND (maintenance OR service* OR assembly OR repair OR training))	Language: English or German; Publication year ≥ 2000; Publication stage: final	1294
Web of Science	TS = (“augmented reality”) AND TS = (tele* OR remote OR collaborat*) AND TS = (maintenance OR service* OR assembly OR repair OR training)	Language: English or German; Timespan: 2000–2020	478
		Records identified by database searching	1,772
		Duplicates removed	202
		Corrected articles	1
		**Total**	**1,569**

**Fig. 2:**
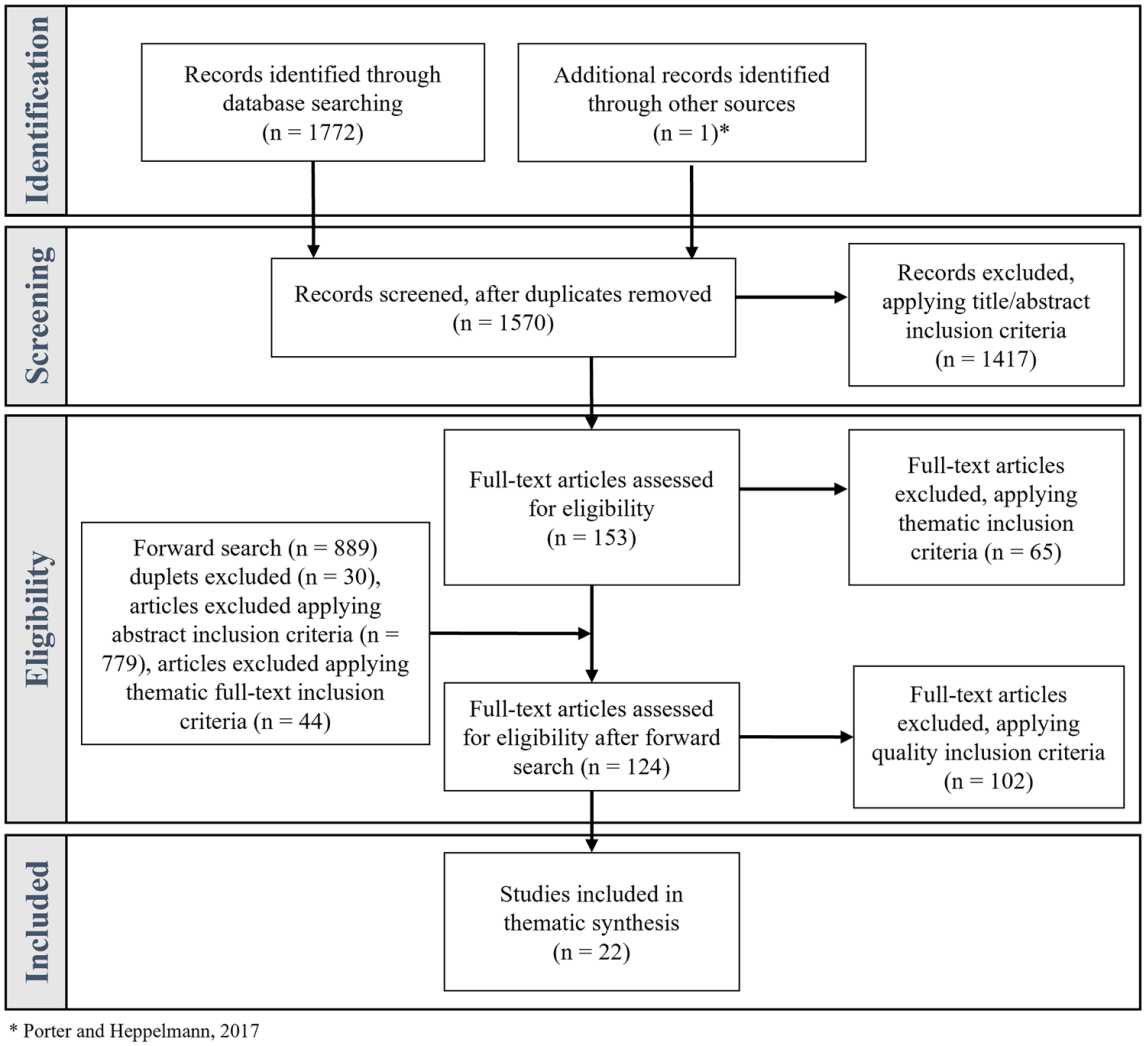
PRISMA flow diagram of the literature screening process (Moher et al. 2009)

After removing duplicates, the search resulted in a total of 1,569 initial hits. On these initial hits, a two-step screening process was applied to identify articles relevant to this research (cf. Fig. 2). The first screening step entailed a title and abstract screening applied using two inclusion criteria: mentioning AR technology and referencing collaboration between at least two physically separated users and/or referencing any technology adoption model. All papers referencing other contexts (e.g., medical, library, and educational) were excluded. The title and abstract screening process resulted in 153 articles, which were forwarded to the second screening step.

The second screening step was a full-text eligibility review using the following two inclusion criteria: descriptions of ARRM technology and maintenance applications. Articles exclusively referring to other industrial applications (e.g., production and quality control) were excluded. Unclear cases were reviewed by a second researcher, and the full-text review for eligibility resulted in 88 papers that were thematically relevant to this research. With these papers, a forward search was performed using both search engines, which resulted in 889 additional hits. The same screening procedure described above was applied again, resulting in 36 additional papers that were thematically relevant to this research and a total of 124 relevant papers.

However, many of these articles were conference papers that only provided superficial descriptions of developed prototypes without serious methodological evaluations. Therefore, only journal papers were included, while conference papers, books, and company reports, among others, were excluded. In doing so, it was ensured that only high-quality, peer-reviewed papers were included in the literature review. Furthermore, review articles were excluded. Table 5 in the Appendix depicts a sample of 22 papers selected for thematic synthesis.

#### Interview study

Supplementing the literature review, empirical data from two ARRM technology suppliers and 14 equipment manufacturers headquartered in German-speaking countries and having tested and/or piloted ARRM technology were gathered through an interview study (i.e., 38 participants from 16 firms). A theoretical sampling method was applied (Eisenhardt and Graebner 2007). The inclusion criteria for the sample were that these companies used similar ARRM configurations, had tested ARRM for a minimum of two months, and had a high diversity of size, products, and level of servitization. For confidentiality, we identified the companies as Case A, Case B, Case C, and so forth (cf. Table 2). Apart from Cases L and N, all companies used ARRM software provided by Case K, a German start-up software supplier with an international clientele. All companies used monocular video HMDs (i.e., Vuzix M300 and/or RealWear HMT-1).

**Table 2:** Characteristics of the interview study sample (n = 16)

**Case**	**Industry**	**Employees**	**Total sales (mm €)**	**Service sales (% of total)**	Position of interviewees (hierarchical level*)	**Interview length (min.)**	Data collection (recording method**)
**A**	Air purification systems	< 50	< 10	~ 5	General manager and owner 1 (E) General manager and owner 2 (E)	81	Group discussion (A)
**B**	Clamping technology	< 1,000	< 100	~ 2	Head of service department (E) Product manager service (U)	70	Group discussion (A)
**C**	Coating machines	< 500	< 250		Head of service sales and repair (E)	75	Individual interview (A)
					Project manager service strategy (M)	58	Individual interview (P)
					Team lead hotline support (M) Team lead internal repair training (M) Team lead of training academy (M) Team lead system installation (M	42	Group discussion (A)
**D**	Drilling machine tools	< 50	n/a	~ 1	Chief executive officer (E)	39	Individual interview (A)
**E**	Finishing machine tools	< 500	< 60	~ 25	Head of service sales (M)	66	Individual interview (A)
					Head of remote support (U)	43	Individual interview (A)
**F**	Food processing	< 10,000	< 3,000	~ 20	Head of customer service (E)	93	Individual interview (A)
**G**	Grinding machine tools	< 1,000	< 200	~ 20	Head of customer care (E)	69	Individual interview (A)
					Project manager service processes (M) Instructor academy (U)	68	Group discussion (A)
**H**	Head-mounted displays	< 250	< 20	n/a	Business development manager (M)	90	Individual interview (P)
**I**	Intralogistics systems	< 5,000	< 1,000	~ 30	Director customer support (E)	68	Individual interview (A)
					Project manager ARRM (M)	72	Individual interview (A)
					Head of hotline support (M)	54	Individual interview (A)
					Team lead 1 customer support (M)	49	Individual interview (A)
					Team lead 2 customer support (M)	41	Individual interview (A)
					Operations and maintenance manager (U)	49	Individual interview (A)
					Maintenance manager (U)	28	Individual interview (A)
					Project manager retrofit (U)	40	Individual interview (A)
					Field service manager (U)	75	Individual interview (A)
J	Material handling systems	< 500	< 100	~ 50	Project lead digital services (M)	100	Group discussion (A)
					Project manager augmented reality (M)		
K	ARRM software	< 50	n/a	n/a	Chief executive officer and founder (E)	63	Individual interview (A)
L	Mechanical and plant engineering	< 20,000	< 4,500	n/a	Director of smart services (E)	156	Individual interview (A)
M	Micro milling machine tools	< 250	< 30	~ 17	Head of service (M)	76	Individual interview (A)
N	Plastic processing machines	< 250	< 25	n/a	Head of mechanical engineering (E)	101	Individual interview (A)
					Project manager (U)		Individual interview (P)
O	Production systems	< 2,500	< 300	~ 22	Project lead of the R&D industry 4.0 (M)	36	Individual interview (P)
					Service manager, Asia (U)	38	Individual interview (A)
P	Valve technology	< 2,500	< 300	~ 7	Head of technical support (M) Digital services consultant (M)	52	Group discussion (A)

*E = Senior executive, M = Service operations manager, U = ARRM user; **A = Audio recorded and transcribed, P = Paper protocol

A total of 38 interviewees from different hierarchy levels, including 11 senior executives, 19 service operations managers, and 8 ARRM users, participated in 32 data collection events (cf. Table 2). Their written informed consent was obtained from legally authorized representatives prior to the study. The data collection events were held in German and lasted an average of 65 min. All individual and group interviews took place between January 2018 and May 2019 and followed a semi-structured interview protocol derived from two focus groups that served as kickoff events for the research project and an earlier literature review that was available back in 2017. The first focus group took place in December 2017 with Cases A, E, G, H, K, and P, and the second focus group took place in January 2018 with Cases D, F, H, K, and N. Focus groups and interviews were audio-recorded and transcribed or paper protocolled, resulting in an empirical database of more than 500 pages of text.

The interview protocol consisted of three parts. Part I sought to understand the business models of the case companies and the professional backgrounds of the interview participants. Part I questions were designed to elicit and assess participants’ viewpoints. Part II sought to understand the motivation of companies to adopt ARRM technologies, and Part III sought to understand their promoting and inhibiting ARRM adoption factors. The full list of questions is available in the appendix of this paper (cf. Table 6).

### Data analysis

The literature review and interview transcript datasets were initially subjected to a deductive descriptive analysis. All papers in the literature dataset were classified according to year, main purpose, industry, application, study type, sample size, and evaluation variable. The case companies in the interview study were classified according to industry, size (i.e., total revenue and employees), business type (i.e., products offered), level of servitization (i.e., share of service revenue and services offered), export ratio, customer segments, and ARRM use cases. The descriptive analysis helped us evaluate the insights drawn from each dataset.

Both datasets were subsequently subjected to thematic analysis, which is an encoding process of qualitative information using explicit codes (Boyatzis 1998). The thematic analysis aimed to identify the promoting or inhibiting items of ARRM adoption, find categorical patterns (i.e., factors of ARRM adoption success) in the data, and analyze the categorical patterns according to the ARRM adoption research framework (i.e., constructs and dimensions). In contrast to the descriptive analysis, the thematic analysis was more inductive in nature. Because coding is a heuristic that does not specify the process steps to follow (Saldaña 2016), in the following paragraph, we disclose the three-cycle coding approach applied to develop the findings of this study.

First-cycle coding aimed to gather simple lists of the items of interest (i.e., benefits, opportunities, challenges, and barriers) raised by interview participants. An inductive structural and holistic coding method was applied as it is particularly appropriate for gathering topic lists (Saldaña 2016). Hence, with respect to the interview transcripts, we conducted line-by-line coding to ensure that we missed no relevant promoting or inhibiting items. With respect to the literature, we coded each aspect that was relevant to our research context in any way, regardless of whether the item was briefly mentioned or supported by the article’s methods.

Second-cycle coding was then performed to check coding consistency at an abstract level and to deduce the first-cycle codes where appropriate. After second-cycle coding, the final list of promoting and/or inhibiting items was available (cf. Online Resource 1). Afterwards, during third-cycle coding, second-cycle codes were analyzed with respect to the research framework discussed in Section 3, which served as the theoretical grounding for this study. To this end, pattern coding (i.e., factoring) was applied (Miles et al. 2014) to categorize the identified items into promoting and inhibiting factors of ARRM adoption success, and to assign the factors to the six constructs of the research framework. The second- and third-cycle coding procedure was applied to all documents by the first author of this paper using NVivo R1 qualitative data analysis software.

## Results

In our explorative qualitative study, we identified 53 promoting or inhibiting items of ARRM adoption and applied the TOE-based research framework (cf. Section 3), resulting in 17 factors affecting the success of ARRM adoption. The findings are listed in Table 3. It is important to stress that the number of items identified in the datasets should not be misinterpreted as an indicator of relevance. Owing to the qualitative nature of the small sample-size interview, the strong technical focus of the literature, and the early stages of ARRM adoption, items identified only once might turn out to be highly relevant. Nevertheless, counting the items allowed us to compare the thematic emphases of the datasets.

**Table 3:** Categorization of augmented reality (AR) remote maintenance (ARRM) technology–organization–environment (TOE) items and factors identified in the literature (n = 22) and interviews (n = 38)

***Third-cycle coding***		***First/second-cycle coding***		***Frequency of mention per study dataset***	
**Construct**	**Factor**	**Items**	**Description / rationale**	**Literature (n = 22)**	**Interview (n = 16)**
**Operational benefits**	***ARRM key feature benefits***	Shared view	Providing a shared view of the workspace (e.g., video stream and/or immersive environment) to raise situational awareness and communicational grounding (especially for remote experts) to reduce misunderstandings, language barriers, or errors	11	9
		Awareness cues	Overlaying reality with virtual content (e.g., 2D or 3D objects, free-hand drawings over video, hand gestures, head pose or eye gaze rays) to raise object awareness and communicational grounding (especially for on-site technicians) to prevent misunderstandings, language barriers, or errors	12	2
		Hands-free communication	Using hands-free mobile computing AR devices so on-site technicians can perform manual work while being remotely instructed (e.g., monocular, or binocular HMDs)	14	9
	***Quantitative service delivery advantages***	Intervention duration	Reducing the time required for remote expert’s service session, regardless of whether they are collaborating with an on-site field service technician colleague, customer’s maintenance personnel, or third-party service provider’s technician	11	6
		Deployment planning	More targeted deployment preparation and scheduling through remote preparation sessions, enabling improved estimate of required skills, spare parts, special tools, and measurement instruments for on-site field service—i.e., more deployment possibilities since technicians with less suitable qualification profiles become deployable	1	7
		Capacity utilization	Balancing variations of capacity utilization (e.g., seasonal peaks), using frontline field service technicians as additional remote expert to support the customer’s and/or third-party service provider’s personnel in situations of short-term capacity shortage or using frontline field service technicians to perform additional services (e.g., remote inspections or application engineering services) in situations of excess capacity	-	3
		First-time fix rate	Increasing on-site first-time fix rates by reducing second deployments for troubleshooting and reworking in cases where the first field service intervention was not successful	2	4
		Initial training	Reducing initial training periods as newly hired service technicians can be deployed to customers earlier with remote expert backup (i.e., shorter period for recruits to accompany experienced technicians)	3	10
		Remote troubleshooting rate	Increasing remote troubleshooting rate by reducing the avoidable travels of field service technicians and other highly scarce experts (e.g., software programmers and trainers)	7	10
	***Qualitative service delivery advantages***	Data reuse	Collecting data through recorded service sessions to map the service history to be reused for training purposes and/or service support tools (i.e., as-build equipment documentation, condition of the installed base for future service cases, or warranty claims and to-build knowledge database or recommender systems)	6	5
		Distributed responsibility	Real-time remote collaboration or service delivery results with less pressure on individuals and lower stress levels (e.g., hectic work in downtime situations reduced, less traveling stress experienced by field service technicians or other experts)	-	10
		Knowledge transfer	Real-time intra- or inter-organizational knowledge transfer and collaboration (including interdisciplinary or intergenerational knowledge transfer—e.g., by real-time consultation with other department’s colleagues from electrical, mechanical, or software engineering, R&D, and IT; knowledge transfer from experienced to novice technicians addresses) challenges of service delivery (e.g., variety and complexity of the installed base, low qualification levels of customer’s or service partner’s personnel, or capacity bottlenecks)	6	7
**Technical challenges**	***AR user experience***	Awareness cues interaction and visualization methods	Providing sophisticated interaction and visualization methods to enable virtual awareness cues (e.g., projecting a remote expert’s hand gestures or avatars and eye- or head-gaze cues) to further improve situational and/or object awareness and communicational grounding	7	-
		Shared view interaction and visualization methods	Providing sophisticated interaction and visualization methods to enable shared views (e.g., immersive environments and solutions to problems such as low image resolution, frozen or shaky video images) to further improve situational and/or object awareness and communicational grounding	6	5
		Virtual content alignment	Recognizing or registering virtual objects at real-world objects (i.e., natural feature-based tracking technology, spatial mapping, and contact analog registration)	13	-
		Head-mounted display (HMD) usability	Lack of industrial maturity under real-life working conditions—i.e., capacity to support hands-free service tasks, while users enjoy the experience (e.g., weight or wearing comfort, field of view, ruggedness, processing power, camera frame rate, battery running time, and intuitive user interface)	12	12
		Health and safety	Occupational safety is affected by HMDs restricting the field of view, by virtual objects blocking the view, or by the head’s proximity to hazardous components—e.g., control cabinet or tool spindle (on-site technicians must approach objects closely with their head for remote experts to achieve situational awareness); users occasionally report eye strain, headaches, and nausea	7	7
	***Data connection***	Data security	Ensuring data security throughout the value creation network to share confidential content or intellectual property (e.g., recorded service sessions, technical drawings, and circuit diagrams)	2	1
		Data transmission	Transmitting data (e.g., video images, virtual content or immersive environments) at a low bandwidth and minimized latency is the basic condition of ARRM-based services (e.g., affected by network architecture or mobile data connection equipment)	9	12
	***Information provision***	Authoring	Providing easy-to-use authoring methods for remote experts—e.g., through the user interface that can be used without CAD or AR-SDK skills or automatic virtual content authoring methods (e.g., sharing ad hoc generated (di)assembly sequences during remote support sessions for spare part installation by combining manual authoring with AI-based data base knowledge)	8	-
		System interoperability	Bidirectional data integration from or to external systems (e.g., SCADA, CAD, CMMS, and ERP) to enrich available information for remote service sessions and/or enable the data processing of previous remote service sessions in other systems	6	1
**Business opportunities**	***Finance***	Revenue	Gaining additional revenue with innovative value-adding service products (e.g., remote operator training and remote consulting services, such as production optimization by application engineers); proactively offering more traditional services to customers since more knowledge about the installed base is available	2	8
		Margin	Achieving a higher margin through savings in resources—c.f., reducing warranty costs and other uncharged hours though operational benefits (e.g., raise in remote troubleshooting or first-time fix rate)	6	8
	***Value proposition***	Global market presence	Covering markets in remote regions with small and/or dispersed installed base (e.g., direct remote support to customers) or delivering service via local third-party service providers	2	4
		Installed base uptime	Reducing customer’s downtime through operational benefits attributed to ARRM service delivery—i.e., reducing customer’s production costs	2	9
		Service portfolio	Offering services that, due to a lack of capabilities or information, were previously not possible, thereby expanding or regionally unifying the service portfolio according to the local market’s expectations	2	6
		Service prices	Reducing the customer’s maintenance costs due to the omission of travel expenses, which are usually passed on to customers	4	10
		Service quality	Service departments are enabled to deliver more efficient or effective services (e.g., field service technicians, including those of subsidiaries and/or third-party service providers) are enabled to perform higher value tasks due to the availability of ad hoc remote assistance and more frequent remote training sessions and/or response or downtimes are decreased due to remote troubleshooting directly with on-site customer’s personnel	5	10
	***Value creation network***	Supply chain disintermediation	Eliminating service value chain stages—i.e., by “cutting off the middleman”, through direct service delivery to equipment users, or by bypassing intermediaries (e.g., dealers or third-party service providers)	-	1
		Supply chain intermediation	Integrating service intermediaries (e.g., third-party service providers) to achieve global market penetration without the need for own local service infrastructure in regions with small and/or distributed installed base	-	1
		Redesigning the organizational structure	Redesigning the service organization by centralizing or decentralizing service knowledge (e.g., by introducing a highly skilled central help desk or regional expertise hubs)	3	1
	***Image***	Innovation capabilities	Proofing or demonstrating innovation capabilities toward customers may be a competitive advantage (i.e., gains in service delivery efficiency by introducing novel technology and gains in customer’s efficiency by introducing novel service products)	-	6
		Customer satisfaction	Improving customer satisfaction though an improved value proposition (i.e., higher service quality, expanded service portfolio, lower service prices, and increased installed base uptime)	3	2
		Service job attractiveness	Increasing attractiveness of service careers due to distributed responsibility (e.g., less pressure and traveling stress); improving the employee’s job satisfaction and leading to recruitment advantages of skilled technicians or trainees by providing proof of the company’s or industry’s innovativeness (i.e., working with novel technology)	-	3
**Management capabilities**	***Adoption management***	Technology configuration	Configuring available technology components to develop a scalable system that can be expanded in terms of capabilities in the future—i.e., performing a critical requirement analysis (e.g., selecting valuable applications feasible already today or only later to define required hardware, software, or data integration capabilities, including the assessment of available technology’s maturity for intended application)	12	2
		Stakeholder involvement	Involving users and cross-departmental stakeholders early in the adoption project (e.g., R&D or IT for data connection and information provision, marketing and sales for roll-out, and medical officer for safety)	2	4
		Process alignment	Aligning or adapting service processes to establish the compatibility of ARRM and the current practice of the company—e.g., ensuring the remote expert’s availability, adapting working procedures to counteract the risk of unintentional knowledge drain to customers or third-party service providers and/or decline in personal contact with customers	2	9
		Internal user acceptance	Actively working toward internal user acceptance—i.e., willingness to use technology by addressing possible user reservation or fears (e.g., transparency, monitoring, privacy, replacements with technology) and identifying ostensible reasons (e.g., technological immaturity is put forward where it is really about fears); relating more to service technicians and less to remote experts	1	11
	***Resource allocation***	Labor	Assigning or providing skilled labor (e.g., experts from other departments too) required for the adoption project (e.g., creating virtual content for extended functionality, authoring and system interoperability)	11	3
		Investment	Committing to invest in technology adoption (i.e., initial hardware or software investment, cost for initial external support by specialized vendors, and employee training)	4	3
		Expenses	Providing a budget for the operating costs of ARRM technology (e.g., software license fees)	2	1
	***Strategic alignment***	Business model	Adapting business models and service-level agreements (i.e., redefining value propositions and revenue models among others) to counteract the risk of decreased traditional revenues from avoidable field service deployments	3	13
		Strategic objectives	Providing clear strategy objectives—defining business opportunities to be seized and how to achieve them (e.g., building up ARRM capabilities inhouse as opposed to outsourcing)	1	-
**Customer Readiness**	***Intellectual property protection***	Installed base access	Access to installed products denied to field service technicians using smart devices due to data security concerns and/or intellectual property protection—i.e., general ban of smartphones or HMDs from customer’s factory premises	-	12
		Outgoing data connection access	Access to on-site (Wi-Fi) networks for external smart devices is denied (e.g., in secluded buildings where mobile wireless broadband does not ensure required data transmission rates) and/or prohibiting outgoing data connection in general	1	9
	***Remote service acceptance***	Involvement	Customer’s inclusion (e.g., IT, manufacturing, or maintenance department) to create on-site preconditions for ARRM-based service delivery—e.g., integrating devices into IT infrastructure to prevent low data transmission rates (e.g., caused by firewalls) or placing AR markers	3	4
		External user acceptance	External user acceptance or willingness to use technology due to the user’s reservation and/or fears (e.g., transparency, monitoring, privacy, and replacements with technology)	-	5
		Willingness to pay	Customer’s willingness to pay value-based prices for remote services—i.e., monetizing greater value of remote troubleshooting (less downtime) compared to the on-site approach that is uncertain for providers	-	5
**External Pressure**	**Skill gaps**	Skills shortage	General lack of qualified technicians available on the labor market (e.g., automation technicians) resulting in extended initial training periods for less suitable recruits and the tie-up of capacity among the few available experts	-	4
		Customers’ skills	Customers increasingly and rarely having their own capable maintenance departments (i.e., collaboration via telephone is challenging, and field service technicians must be deployed for cases that are less complex)	-	1
		Service partners’ skills	Service partner’s skills often not meeting increased field service demands (e.g., variety and complexity of installed base and low qualification levels of customer’s maintenance personnel), especially in markets with small and/or dispersed installed base, where the incentive to participate in regular training is low	-	2
	**Governmental regulation**	Climate crisis	Reducing environmental impact though dematerialized value creation (e.g., decline in intercontinental travels and spare part shipments and returns) as a measure against ever stricter requirements or higher costs of service delivery (e.g., CO_2_ tax or certificate trading)	1	-
		COVID-19*	Travel restrictions introduced during the COVID-19 pandemic forcing companies to deliver complex services remotely (e.g., machine installation, factory acceptance tests, and application engineering)	-	-

*Notes:* *Not identified in the dataset but supplemented because it seems to be a promoting factor of ARRM adoption

The analysis uncovered 21 items within the technological dimension, categorized into six technological factors that promote or inhibit adoption success. Of these six factors, three (i.e., *ARRM key feature benefits*, *quantitative service delivery advantages*, and *qualitative service delivery advantages*) were attributed to the construct of operational benefits and were expected to have a promoting effect on ARRM adoption in the adopting organization. The other three factors (i.e., *AR user experience*, *data connection*, and *information provision*) were attributed to the construct of technical challenges and are expected to have inhibiting effects. The interview study added items of *capacity utilization* and *distributed responsibility*. On the other hand, the literature review uncovered items of *virtual content alignment* and *authoring* that were not observed in the interview study.

Within the organizational dimension, we uncovered 22 items relevant to ARRM adoption. These were categorized into seven organizational success factors, of which four (i.e., *value proposition*, *value creation network*, *finance*, and *image*) were attributed to the business opportunity construct and three (i.e., *adoption management*, *resource allocation*, and *strategic alignment*) to management capabilities. All organizational factors are expected to have promoting effects on ARRM adoption. The interview study uncovered four new items that were not yet reflected in the ARRM literature (i.e., *supply chain disintermediation*, *supply chain intermediation*, *innovation capabilities*, and *service job attractiveness*). Although it is of little surprise, given the strong engineering focus of the ARRM literature (Breitkreuz et al. 2022), we found that organizational factors were less reflected in the literature (Herterich et al. 2015; Masood and Egger 2019).

With respect to the environmental dimension, our analysis uncovered a total of 10 items, categorized into four environmental factors, of which two (i.e., *intellectual property protection* and *remote service acceptance*) were attributed to the customer’s readiness construct and the other two (i.e., *skills gap* and *governmental regulation*) to external pressure. Customer readiness factors are expected to have inhibiting effects, whereas external pressure factors are expected to have promoting effects on ARRM adoption. The interview study added six environmental items (i.e., *installed base access*, *external user acceptance*, *willingness to pay*, *skills shortage*, *customers’ skills*, and *service partners’ skills*) to those acquired from the literature review. On the other hand, the literature review added a *climate crisis* item. This shows that our methodology has contributed new insights into the environmental aspects of industrial AR adoption, an area in which very little attention has been focused thus far (Egger and Masood 2020). As expected, the insights from the interview study disclosed organizational, environmental, and product–service system-specific aspects, whereas the literature review revealed technical aspects more comprehensively.

## Discussion

In this section, we place the findings of our study in the context of the comparable literature introduced in Section 2.2 and discuss their similarities and differences. The extent to which success factors were recognized in the related works is illustrated in Table 4.

**Table 4:** Augmented reality (AR) remote maintenance (ARRM) adoption success factors identified in our study versus related works

Construct	Success factors	Related works	
		Fully reflected	Partly reflected
OB	ARRM key features	Jalo et al. (2018)	Rapaccini et al. (2014), Porter and Heppelmann (2017)
	Quantitative service delivery advantages	Si2 Partners (2018)	-
	Qualitative service delivery advantages	-	Rapaccini et al. (2014)
TC	AR user experience	-	Jalo et al. (2022), Masood and Egger (2019), Porter and Heppelmann (2017), Rapaccini et al. (2014), Si2 Partners (2018)
	Data connection	-	Rapaccini et al. (2014)
	Information provision	-	Jalo et al. (2022), Porter and Heppelmann (2017), Si2 Partners (2018)
BO	Finance	-	Jalo et al. (2022), Porter and Heppelmann (2017), Rapaccini et al. (2014)
	Value proposition	-	Si2 Partners (2018)
	Value creation network	-	Si2 Partners (2018)
	Image	-	Porter and Heppelmann (2017), Si2 Partners (2018)
MC	Adoption management	Masood and Egger (2019)	Jalo et al. (2018), Jalo et al. (2022), Rapaccini et al. (2014), Si2 Partners (2018)
	Resource allocation	Jalo et al. (2022), Si2 Partners (2018)	-
	Strategic alignment	-	Porter and Heppelmann (2017), Rapaccini et al. (2014), Si2 Partners (2018)
CR	Intellectual property protection	Si2 Partners (2018)	Rapaccini et al. (2014)
	Remote service acceptance	-	Jalo et al. (2018), Jalo et al. (2022)
EP	Skill gaps	-	-
	Governmental regulation	-	-

*OB* operational benefits, *TC* technical challenges, *BO* business opportunities, *MC* management capabilities, *CR* customer readiness, *EP* external pressures

### Technological factors

#### Operational benefits

Other TOE studies on industrial AR did not observe operational benefits—i.e., the relative advantage of industrial AR compared to previous approaches (Jalo et al. 2022; Masood and Egger 2019). This might be due to the fact that the broad range of technologies and use cases covered in those studies (e.g., AR/VR/XR in sales, logistics, and teaching) made it difficult to describe the relative advantages of an innovation compared to the replaced approach. However, operational benefits indicating the relative advantage of an innovation were among the best predictors of technology adoption (Arnold and Voigt 2019; Hsu et al. 2006; Jeyaraj et al. 2006; Kuan and Chau 2001; Mangula et al. 2015). Moreover, our results are in line with previous research on ARRM in the service context (Jalo et al. 2018; Porter and Heppelmann 2017; Rapaccini et al. 2014; Si2 Partners 2018).

Some findings of our study are worth mentioning. For example, the importance of the hands-free communication feature of HMDs was controversial among the case companies in our study. Although some interviewees were convinced that the hand-free feature was indispensable, others preferred handheld devices (e.g., smartphones or tablets) owing to their superior computational power and availability. This agrees with other recent studies that found that on-site technicians often prefer using handheld devices (Jalo et al. 2022; Marques et al. 2022; Si2 Partners 2018). However, the ARRM engineering research community has recently developed systems that exclusively use HMDs (Fang et al. 2020; Mourtzis et al. 2020; Wang et al. 2020). The preferred mobile AR platform seems to be a matter of the specific use case. For example, when guiding inexperienced on-site personnel, such as novice field service technicians during initial training periods or customer’s machine operators, the hands-free aspect is considered crucial because the sessions require guided hands-on training. On the other hand, when experienced field service technicians consult remote experts to discuss complicated failures, handheld devices are often preferred. This indicates that different use cases require different ARRM configurations.

#### Technical challenges

The technical challenges (i.e., AR user experience, data connection, and information provision) are only partly reflected in related studies (Jalo et al. 2022; Masood and Egger 2019; Porter and Heppelmann 2017; Rapaccini et al. 2014; Si2 Partners 2018). In this regard, our methodology enhances state-of-the-art industrial AR adoption.

The most pressing technical challenge hampering the AR user experience is the usability of HMDs in terms of weight, wearing comfort, field of view, ruggedness, processing power, camera frame rate, battery running time, and intuitive user interface. HMDs’ immaturity is well documented in the ARRM literature (Fang et al. 2020; de Pace et al. 2019; Piumsomboon et al. 2019) and has been identified as a significant inhibiting factor of adoption success (Jalo et al. 2022; Masood and Egger 2019; Si2 Partners 2018). Overall, the case companies (apart from Cases D and P) of our interview revealed that the RealWear HMT-1 HMD is mature enough for industrial service use.

ARRM is not only a collaboration tool used to facilitate knowledge transfer, but it is also an information-gathering tool that enables data reuse (del Amo et al. 2020; Lamberti et al. 2014). In industrial practice, customers and equipment manufacturers regularly disagree on the responsibility for failures, especially during warranty periods. Therefore, more efficient approaches to documenting service interventions are beneficial to equipment manufacturers. In this context, the information provision success factor, especially the system interoperability indicator, is relevant. The low recognition rate of the factor in our interview study can be explained by the fact that the ARRM systems used by the case companies were standalone systems that were not integrated into the organizations’ existing IT infrastructure, and sophisticated authoring tools were not available during testing or piloting periods. As a result, issues related to authoring and system interoperability were probably outside the scope of the interviewees’ experiences. However, despite the low recognition of this factor, information provision is likely to be an important factor going forward because, in industrial practice, ARRM systems must be compatible and integrated with existing infrastructures (Jalo et al. 2022; Masood and Egger 2019). Moreover, remote service delivery compatibility and integration can provide easy-to-use information provision tools for users, while granting them access to reusable data from previous service interventions.

Data connection was not observed as a success factor in comparable TOE-based adoption studies of industrial AR adoption when a wide range of applications and use cases was investigated (Jalo et al. 2022; Masood and Egger 2019). However, our results are in line with AR studies in the context of remote service delivery (Runji et al. 2022; Si2 Partners 2018), and other remote service technologies for product–service systems (Klein et al. 2018). From the product–service system perspective, reliable data transmission is a pressing issue. Our case companies pointed out that it would be favorable to remain independent from customers’ on-site network infrastructures via the use of mobile data connection equipment. However, customer production facilities are often located in remote rural areas. Even if mobile data services are available, the signal strength will be weak inside the facility. Case companies I and M reported issues with customers in remote regions (e.g., Siberia), where even their fully functional local networks were insufficient to transmit video. Moreover, secure communication protocols and data security throughout the entire service delivery network are required (Mourtzis et al. 2018; Rapaccini et al. 2014).

### Organizational factors

#### Business opportunities

Success factors related to business opportunities were not observed in other TOE-based adoption studies on industrial AR (Jalo et al. 2022; Masood and Egger 2019). Because business opportunities are a form of relative advantage, the same explanation can be putted forward as with operational benefits: the broad range of technologies and use cases covered in those studies (e.g., AR/VR/XR in sales, logistics, and teaching) makes it difficult to identify concrete business opportunities. However, in related studies that focused on the service context, the business opportunities identified in our study were reflected to some extend (Jalo et al. 2018; Porter and Heppelmann 2017; Rapaccini et al. 2014). Altogether, we were able to identify 12 business opportunity items categorized into four success factors, indicating the potential of ARRM for transforming business models and organizational structures. Thus, we argue that business opportunities are an important motivator for senior executives to adopt ARRM and, consequently, to support adoption initiatives.

Interestingly, in comparable studies, the cost-saving aspect of reductions in travel is dominant (Porter and Heppelmann 2017; Rapaccini et al. 2014; Si2 Partners 2018). Our study, on the other hand, found that cutting out traveling per se is not a concern for equipment manufacturers because traveling costs are usually passed on to customers and are thus pass-through costs. Only two companies (Cases D and P) referred to cost savings as the main driver of ARRM adoption. However, it is worth mentioning that both companies were at low servitization levels and struggled to charge for services in the first place. The other case companies were particularly interested in avoiding specific travels that could not be charged, such as deployments during warranty periods (i.e., improving the remote troubleshooting rate) or second deployments due to failed first-service interventions (i.e., improving the first-time fix rate).

#### Management capabilities

Adoption management is also observed in comparable studies suggesting that technology configuration, process alignment, and stakeholder involvement are significant for adoption success (Jalo et al. 2018, 2022; Masood and Egger 2019; Rapaccini et al. 2014). Moreover, companies seem to underestimate the efforts required for successful ARRM adoption, such as managing the lack of acceptance, implementing the right processes, and adapting business models (Si2 Partners 2018).

Regarding internal user acceptance, our results are not clear-cut. Reservations in adopting ARRM relate more to the on-site service technicians and less to the remote experts. Other research suggests that resistance to this technology is more of an issue with older employees (Jalo et al. 2022). Moreover, Cases F and M shared that the real reason for low on-site service technician acceptance is often fear (e.g., transparency, monitoring, privacy, and replacements with technology), rather than the ostensible reasons put forward by users (e.g., customers not wanting remote service or a lack of HMD’s maturity). The ambiguity of this result is also reflected in previous studies. In a quantitative survey, user acceptance did not correlate with AR adoption success (Masood and Egger 2019); however, they reported that their qualitative investigation suggested that it was relevant for companies. Therefore, change management and user involvement seem important in mitigating possible user resistance (Jalo et al. 2022). However, many companies seem to have problems managing this change process (Si2 Partners 2018).

Therefore, apart from operational adoption management, top management support is required. This is particularly important as its lack will curtail many other success factors (Klein et al. 2018), including resource allocation and strategic alignment of the ARRM adoption process (Jalo et al. 2022; Porter and Heppelmann 2017; Rapaccini et al. 2014; Si2 Partners 2018).

Adopting remote service technologies impacts and disrupts equipment manufacturers’ business models (Marcon et al. 2022), especially when work is shifted to customers (Si2 Partners 2018). In our study, senior executives expressed concerns that traditional service revenues from field service deployments would decrease when more service calls could be solved remotely. To counteract this decrease in service revenue, business models must be adapted as ARRM is seen as an enabler to transform a product-oriented product–service system into a use-oriented product–service system (Mourtzis et al. 2017b).

### Environmental factors

#### Customer readiness

Considering that ARRM is an inter-organizational technology, customer readiness is expected to strongly affect successful ARRM adoption (Oliveira and Martins 2010; Zhu et al. 2003). According to Jalo et al. (2022), AR can still be used in internal operations, even if customers are not yet ready. However, this does not apply to ARRM as the customers are embedded into the service delivery concept, even when only granting access to equipment. Moreover, intellectual property protection in sensitive customer industries and a lack of on-site data connections are sometimes knockout blows for ARRM-based service delivery (Si2 Partners 2018). Additionally, customers must realign their own infrastructures for remote service delivery (Marcon et al. 2022). Therefore, owing to its inter-organizational nature, a lack of customer readiness can be a huge barrier to ARRM adoption.

#### External pressure

Compared with other industrial AR adoption studies, we did not observe competitive pressures and external support as success factors for ARRM adoption (Jalo et al. 2022; Masood and Egger 2019; Si2 Partners 2018). Instead, skill gaps and governmental regulations were identified as sources of external pressure. By identifying these factors, our study contributes to the recent call for future research on environmental factors, which are even less reflected in the literature than organizational factors until now (Egger and Masood 2020; Masood and Egger 2019).

When the interview study participants were asked why they were interested in ARRM in the first place, the most common answers related to their skill gaps in the backdrop of an installed base that is growing more complex and variational over time. This contrasts with other studies that found that operational benefits, cost reductions, and customer pressure for better performance were the main drivers (Si2 Partners 2018).

Skill gaps are characterized by several dimensions. First, a general skill shortage in the European labor market is evident, meaning that equipment manufacturers presently hire less-qualified technicians (Herterich et al. 2015), which extends initial training periods considerably. The director of customer support at the Case I company spoke of a “talent war for qualified technicians” who are willing to sign up for field service jobs, which requires people who are highly stress-resistant, willing to travel internationally, eloquent, and tolerant of working hours. Apart from these, the baby boomer retirement wave is imminent, which threatens to result in the loss of vast service knowledge, especially regarding the older machinery of the installed base. Second, equipment manufacturers have reported that they are being confronted with increasingly less qualified maintenance personnel at the customers’ end, which makes telephone support and remote failure diagnosis more difficult. Third, many equipment manufacturers collaborate with external service partners who service parts of their installed base (Marcon et al. 2022). These service partners’ technicians require regular training sessions. However, due to the effort and cost of sending field service technicians to equipment manufacturers’ training academies, service partners are often less qualified than desired.

Moreover, we argue that governmental regulation is an environmental factor that pressures equipment manufacturers to adopt ARRM. Although the impact of COVID-19 was not reflected in the datasets analyzed due to the time of data collection, we came across several cases in which industry partners reported that they prioritized ARRM adoption during the pandemic. The imposed travel restrictions forced the issue, even with the installation of the delivered machinery (Cavaleri et al. 2021; Li et al. 2022; Wuest et al. 2020). Thus, we assume that COVID-19 initially boosted ARRM adoption initiatives. Additionally, ARRM-based product–service systems can offer a dematerialized solution that minimizes environmental impact (Mourtzis et al. 2017b; Reim et al. 2015). Given the fact that industry faces ever-stricter governmental sustainability requirements, we argue that novel remote service offerings that counteract intensive travel regimes might have a promotional impact on ARRM adoption.

## Conclusions

The aim of our study was to establish ARRM adoption success factors in the specific context of product–service systems. To do so, we employed an exploratory qualitative study design utilizing a systematic literature review of 22 relevant articles and an empirical interview study with 38 interviewees from 16 companies. Based on our analysis, the research question of this study, “What are promoting and inhibiting ARRM adoption factors when considering the specific challenges of industrial product–service systems provision?” was answered as follows. The presented study allowed us to propose 17 technological, organizational, and environmental success factors for ARRM adoption. Furthermore, 12 of these are expected to have a promoting impact on ARRM adoption, and the other five are expected to have an inhibiting impact. Moreover, we were able to contribute 53 specific items, operationalizing these success factors.

### Theoretical contributions

**Fig. 3:**
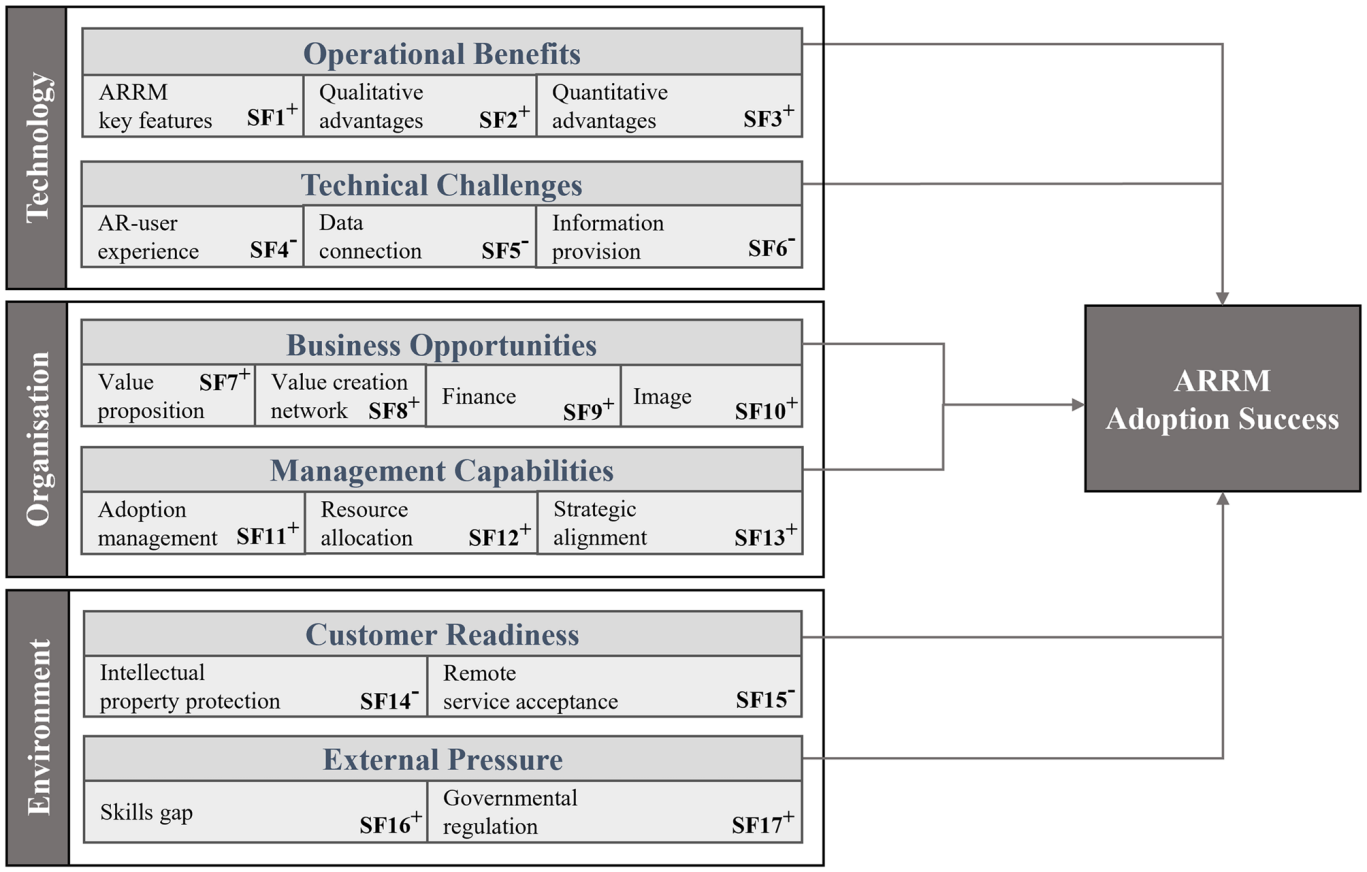
Augmented reality (AR) remote maintenance (ARRM) adoption model—SF*n* (the *n*^th^ success factor)

To the best of the authors’ knowledge, the paper at hand is the first attempt to analyze the success factors of ARRM adoption in support of product–service systems. Thus, this paper contributes a novel ARRM adoption model based on the TOE framework. The model and the corresponding effects of each factor are illustrated in Fig. 3. There, we can observe the promoting (+) and inhibiting (-) effects. Our study is novel in several ways. First, we systematized prior findings that were spread across the latest engineering-focused ARRM literature. Second, we added new success factors that were not or somewhat accounted for in comparable industrial AR adoption studies. Third, we operationalized and clarified the success factors for the specific inter-organizational ARRM adoption context.

Although the available ARRM literature provides insights into engineering aspects from the perspective of prototype development, and earlier adoption studies identify the success factors of a wide range of industrial AR use cases and technologies, this paper viewed industrial AR adoption from a holistic inter-organizational perspective of ARRM in support of product–service systems. Therefore, the insights from the engineering-focused research were contextualized into the application context of product–service systems, making the information accessible to a much broader range of research communities, including servitization.

By analyzing industrial AR adoption in this way, we have clarified how ARRM adoption is more complex than other internal AR applications because external agents to the adopting company must be involved in the adoption process. As a result, we discovered relevant environmental success factors (i.e., skill gaps and governmental regulations) that were not previously observed as success factors or inhibitors. Moreover, our interview study added 12 specific items to operationalize the adoption of ARRM success factors more precisely than the available body of literature. By filling in these gaps, we show that our more well-rounded methodology enhances the recent state-of-the-art. Moreover, we have answered recent calls for research on organizational and environmental factors (Egger and Masood 2020; Herterich et al. 2015; Jalo et al. 2022; Masood and Egger 2019), and by adding and clarifying these, we have created value for technology and engineering scholars and adopters who will soon be developing enhanced prototypes. Moreover, we set the groundwork for the potential technological improvements thereof. For example, our study found that practical users tend to favor handheld devices over HMDs, whereas the engineering research community remains very focused on HMDs.

### Practical contributions

The 53 specifically identified items that led to 17 technological, organizational, and environmental success factors are expected to assist service operations managers and senior executives in developing ARRM adoption strategies. On the one hand, understanding the promoting factors will help them develop novel service offerings and business models. On the other hand, understanding the inhibiting factors will help them mitigate risks and overcome barriers associated with ARRM adoption.

Our study revealed that the managers of our cases perceived ARRM as a standalone tool. However, if ARRM can be integrated into existing IT infrastructures (e.g., ticketing or ERP), the data gathered can be reused for training, documentation, and quality control. Thus, only integrated ARRM systems can unfurl their full power with respect to the potential operational benefits and business opportunities. Therefore, managers should contextualize system interoperability when pursuing ARRM projects, which will undoubtedly make adoption more complex. However, although ARRM adoption may disrupt existing processes and business models, managers should not consider this a threat; it is a remarkable opportunity to smartly transform business models so that their multifold processes can survive in the present and future. Moreover, it is an opportunity to transform product–oriented product–service system into use-oriented product–service system.

In this case, professional project management and top-level support will be required to help firms push through tough changes. It is feared that although most executives recognize the benefits and opportunities arising from ARRM, they underestimate the effort and attention to detail required for successful adoption. Overall, we assert that ARRM adoption requires more management attention than is currently provided.

### Limitations and future research

The present study is not without limitations. First, it is worth noting that because adoption research is multidisciplinary, extensive, and often contradictory, it is not possible to develop a monolithic, all-world model, as doing so would include an unmanageable number of interrelated constructs (Grover 1993). Therefore, practically every adoption study suffers from the limitation that potential constructs and factors likely exceed the manageable number of constructs and factors used within empirical technology adoption studies (Sila 2013). Second, our research methodology has limitations that create opportunities for the future. The qualitative interviewing of a limited number of companies always raises the question of the results’ generalizability (Voss et al. 2002). Our study does not overcome the generalizability problem, but the novel combination of empirical interview results with the findings from the most applicable literature provides a nuanced and therefore well-rounded model. The most obvious avenue for future research is testing the proposed ARRM adoption model against larger samples using statistical methods to detect and prioritize critical success factors. To this end, our ARRM adoption model can be used to develop a superior data collection instrument that will lead to its validation in multiple fields. Uncertainty and sensitivity analyses are also needed so that the myriad of future literature will be more practical and broad-minded.

Apart from this obvious avenue, it seems promising to analyze the impact of specific ARRM applications on ARRM adoption success factors. For example, whether the remote collaborative service session takes place between a remote expert and their field service technician colleague or a customer’s and/or service partner’s maintenance technician might affect the relevance of the established ARRM adoption success factors. Moreover, because difficulties in information provision, transport, and processing may pose challenges for adoption and decrease the relative advantages of this innovation, the information needs of ARRM-based product–service systems must be fully extrapolated, and the necessary technological integrations needed to address the multifarious industrial configurations need specifying. These opportunities provide promising avenues for advancing the field. Additionally, analyzing the role of ARRM for business models is expected to be a theoretical hotbed of new activity. For example, an analysis of the extent to which ARRM is an enabler of servitization.

### Electronic supplementary material

Below is the link to the electronic supplementary material.

Supplementary file1 (XLS 37 KB)

## Data Availability

Aggregated data is available as supplementary material at 10.1007/s12063-023-00356-1.

## Appendix

**Table 5:** Characteristics of the papers selected for literature review (n = 22)

#	Author	Application	Study type	Sample	Evaluation variables
1	Adcock and Gunn (2015)	Guidance (placing magnets or drawing shapes on a whiteboard)	Lab experiment	24 university students or staff members	Task completion time, collaborative efficiency, instructiveness, and overall preference
2	Aschenbrenner et al. (2019)	Maintenance (servo amplifier replacement of switch cabinet)	Lab experiment	50 university students 110 technician apprentices	Task completion time, errors, usability, and workload (NASA TLX)
3	Bottecchia et al. (2009)	Maintenance (unspecified assembly task)	n/a	n/a	n/a
4	Choi et al. (2018)	Maintenance of a coffee machine, robot manipulator, and 3D printer	Lab experiment	58 participants	Task completion time, task accuracy, and usability (e.g., usefulness, ease of use, and ease of learn)
5	de Pace et al. (2019)	Training (3D-printed robot hand assembly)	Lab experiment	20 university students, additional tests without using the audio channel with six volunteers	Task completion time, errors, and usability
6	Fang et al. (2020)	Maintenance (e.g., server rack cabling)	Lab experiment	30 participants without background in repairs	Task completion time and errors
7	del Amo et al. (2020)	Fault diagnosis of an aircraft’s fuel hatch	Lab experiment, interviews	30 university students and 8 real-life technicians	Task completion time, errors, usability, and feasibility
8	Fernández-Caramés et al. (2018)	Maintenance (unspecified task at a bending machine)	Industrial case study	Case company Navantia	Response latencies for different data transmission file sizes and percentage of successful transmissions
9	Ferrise et al. (2013)	Motor replacement of a washing machine	Lab experiment	20 university students	Usability
10	Huang and Alem (2013)	Guidance (LEGO assembly task)	Lab experiment	20 participants	Usability and ability to support collaborator's mobility
11	Koteleva et al. (2020)	Maintenance (disassembly of a vertical electric centrifugal pump)	Lab experiment	16 participants	Task completion time
12	Lamberti et al. (2014)	Customer support or training (e.g., tool-holder replacement)	Lab experiment	24 university students	Task competing time and errors
13	Le Chenechal et al. (2019)	Navigation in workspace and guidance (e.g., motherboard assembly)	Lab experiment	14 participants (number of pilot study participants not disclosed)	Task completion time, mean distance, precision, workload, and usability (e.g., eyestrain and perceived naturalness)
14	Masood and Egger (2019)	Quality, maintenance, support, design, training, assembly, and picking	Survey	84 participants (incl. 15 C-suite members, 23 project leads, 22 application or innovation managers)	System configuration, hardware readiness, compatibility, user barrier, organizational fit, and external support
15	Mourtzis et al. (2017b)	Customer support (valve lubrication)	Industrial case study	1 service case: manufacturer serves distant customers	Maintenance duration, cost, and usability
16	Mourtzis et al. (2018)	Customer support (gear grease change)	Industrial case study	5 customers of ARRM provider	Satisfaction with the remote expert, maintenance duration, cost, and overall satisfaction with remote service
17	Mourtzis et al. (2020)	Customer support	Industrial case study	n/a	Operating and maintenance costs, downtime, and time per maintenance
18	Ong and Zhu (2013)	Maintenance (unspecified task)	n/a	n/a	n/a
19	Piumsomboon et al. (2019)	Identification and manipulation of virtual objects	Lab experiment	32 participants	Task completion time, rate of mutual gaze, number of hand gestures, physical movement, distance between collaborators, usability, social presence
20	Porter and Heppelmann (2017)	Field service and customer support	Industrial case study	Case companies: KPN, Lee Company, and Xerox	Repair errors or quality, first-time fix rate, service job efficiency, remote troubleshooting rate, costs, and ROI
21	Rapaccini et al. (2014)	Field service	Industrial case study (survey and interviews)	Case company Océ Italia (survey: 65 field technicians, interviews: 1 service director, 1 remote expert, 2 technicians)	Perceived usefulness, ease of use, and ability to support field service delivery
22	Wang et al. (2020)	Training (water pump assembly)	Lab experiment	28 university students	Task completion time, errors, usability, and overall preference

**Table 6:** Interview protocol of the interview study

**Part**	**Areas of investigation**	**Objective(s)**	**Guiding questions**
I	Company and participant information	To understand the business model and the structure of the service organization, and to be able to assess the study participant's views	• Please describe your professional career? Please describe your current role in your company (area of responsibility/to whom do you report)? • *Please describe your company’s business model? Please describe the role of the service business for the entire business model? Please explain how important the service business is for your company. How does service contribute to the revenue/profit of your company? • *To which customer group(s) do you sell your products/services? What different requirements do your customer groups have (product vs. service business)? • **Which product group (machines) do you service • To which regional markets are you selling products (machines)/services (please quantify)? • *What are the "must-have" services (vs. differentiation potential)? What is the value proposition to your customers? How do you assess your customers’ maintenance know-how (do they typically have own maintenance departments)? • *To what extent does your company sign service agreements? Do these agreements include availability commitments? If so, what exactly is promised (reaction times, availability of technicians, equipment availability etc.)? Do you offer operator models / pay per use models? • Please describe the organization of your service business (central vs. decentral). Does your company follow an active service marketing approach? Do you collaborate with dealers and/or third-party service providers? • Please describe the organizational structure of the remote customer support unit? How large is the part of the installed base for which you offer remote support (remote access to machine control, condition monitoring etc.)? What is your remote troubleshooting rate? • What do you consider to be the most important trends/development paths for the service business/customer support in your industry? Please outline your company’s digitization strategy for the service business (if there is one)?
II	Adoption drivers	To understand the motivation to adopt ARRM, i.e., the pain points of the service business unit	• What is the main driver for initiating an ARRM adoption project? What are other major pain points of your service business unit? • When did you start testing/piloting ARRM? What phase are you currently in: test level, pilot, operational (with business model)?
	Use cases and functional scope	To understand the intended application of ARRM	• Please describe your use case(s) (tested/piloted vs. operational, if applicable)? • Have you considered other use cases, but discarded it later? If so, for what reason? • Please describe the functional scope of the system used. Is there any required function that the system does not have (also regarding discarded use cases)?
III	Benefits and opportunities	To understand factors promoting ARRM adoption	• What is enabled through ARRM that was not possible before (shop floor vs. business perspective!)? • What operational benefits arise from ARRM (your own company vs. customers)? • What business opportunities arise from ARRM? • **What benefits do you personally find in the technology for your daily work (can you do your work easier, faster, with higher quality)? • Does ARRM enable innovative service products that were not possible before? • Are you able to explore new customer groups that you could not reach before (business process vs. business model innovation)?
	Challenges and barriers	To understand factors inhibiting ARRM adoption	• Can you think of any general disadvantages of using ARRM (under the assumption that the system works perfectly)? • How do you assess the technical maturity of ARRM? What problems did you encounter? • What peripheral problems did you encounter, which do not concern the ARRM system directly? • Which organizational problems/challenges did you encounter (own organization, customers, dealers, third-party service providers)? • *What do users think about ARRM? How is ARRM received by the users (office helpdesk experts vs- field service technicians)? **How do you assess the usability of the system? • Which other systems should be integrated with ARRM? **What additional information would make your daily job easier? • Have you encountered / do you anticipate any structural problems in your company which might complicate the adoption process?

* Question only asked to senior executives and service operation managers

** Question only asked to users
